# Supplementary material for: TRPM8 contributes to sex dimorphism by promoting recovery of normal sensitivity in a mouse model of chronic migraine
Source: Nat Commun. 2022 Oct 22;13:6304. doi: 10.1038/s41467-022-33835-3 (PMC9588003; doi:10.1038/s41467-022-33835-3)
Supplement: Supplementary file 1 — Supplementary Information [file 41467_2022_33835_MOESM1_ESM.pdf]

Supplementary Information for:

TRPM8 contributes to sex dimorphism by promoting recovery of normal sensitivity in a mouse model of chronic migraine

David Alarcón-Alarcón[1&], David Cabañero[1&*], Jorge de Andrés-López[1], Magdalena Nikolaeva-Koleva[1], Simona Giorgi[1], Gregorio Fernández-Ballester[1], Asia Fernández-Carvajal[1#*], Antonio Ferrer-Montiel[1#*]

[1] *Instituto de Investigación, Desarrollo e Innovación en Biotecnología Sanitaria de Elche (IDiBE), Universidad Miguel Hernández de Elche, Spain*

[&],[#]: Contributed equally

Materials &Correspondence:

[*] Authors for correspondence:

Prof. Antonio Ferrer-Montiel: aferrer@umh.es

Prof. Asia Fernández-Carvajal: asia.fernandez@umh.es

Dr. David Cabañero: dcabanero@umh.es

Content:

- **Supplementary Figures 1-6 and captions**
- **Supplementary Methods**
- **Supplementary Statistical Results**

Supplementary Figure 1

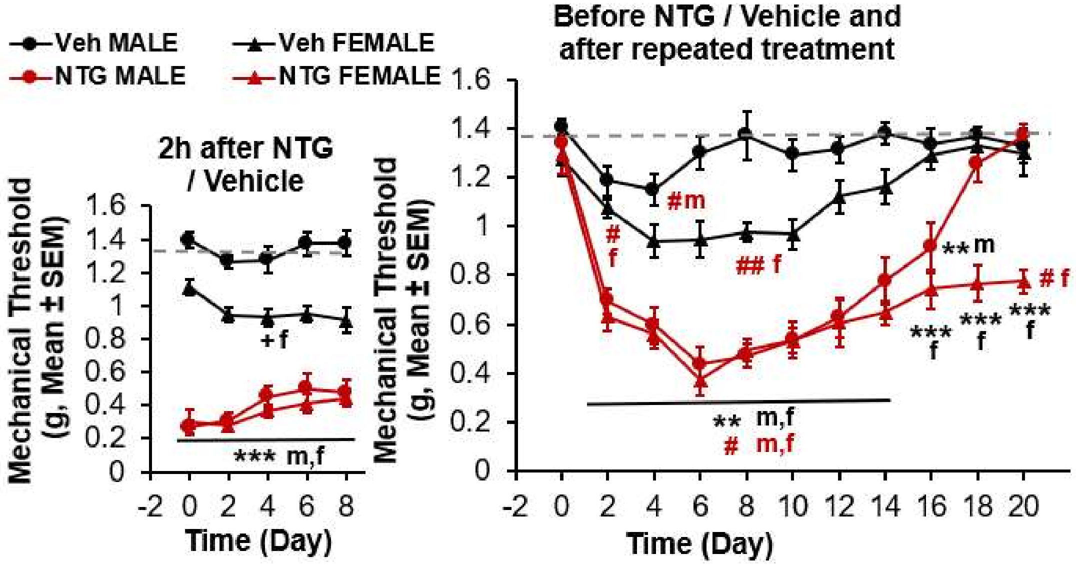

Supplementary Figure 1. Nitroglycerin and a vehicle with high percentage of ethanol and propylene glycol induce mechanical sensitization. Left panel**:** Nitroglycerin (red) 10 mg/kg dissolved in 10% ethanol and 20% propylene glycol induces similar acute mechanical hypersensitivity in males and females, and this vehicle (black) induces a selective acute sensitization in females. Right panel: Chronic nitroglycerin induces persistent hypersensitivity exclusively in females, and a transient hypersensitivity is observed in males and females treated with this vehicle. Data are ± S.E.M. **p<0.01, ***p<0.001 vs vehicle. #p<0.05, ##p<0.01 vs baseline. +p<0.05 vs day 0. 2-way RM ANOVA. n=6 per condition. NTG, nitroglycerin. Veh, Vehicle. f, female. m, male. Source data are provided in Source Data file, statistical results in Supplementary table 6.

Supplementary Figure 2

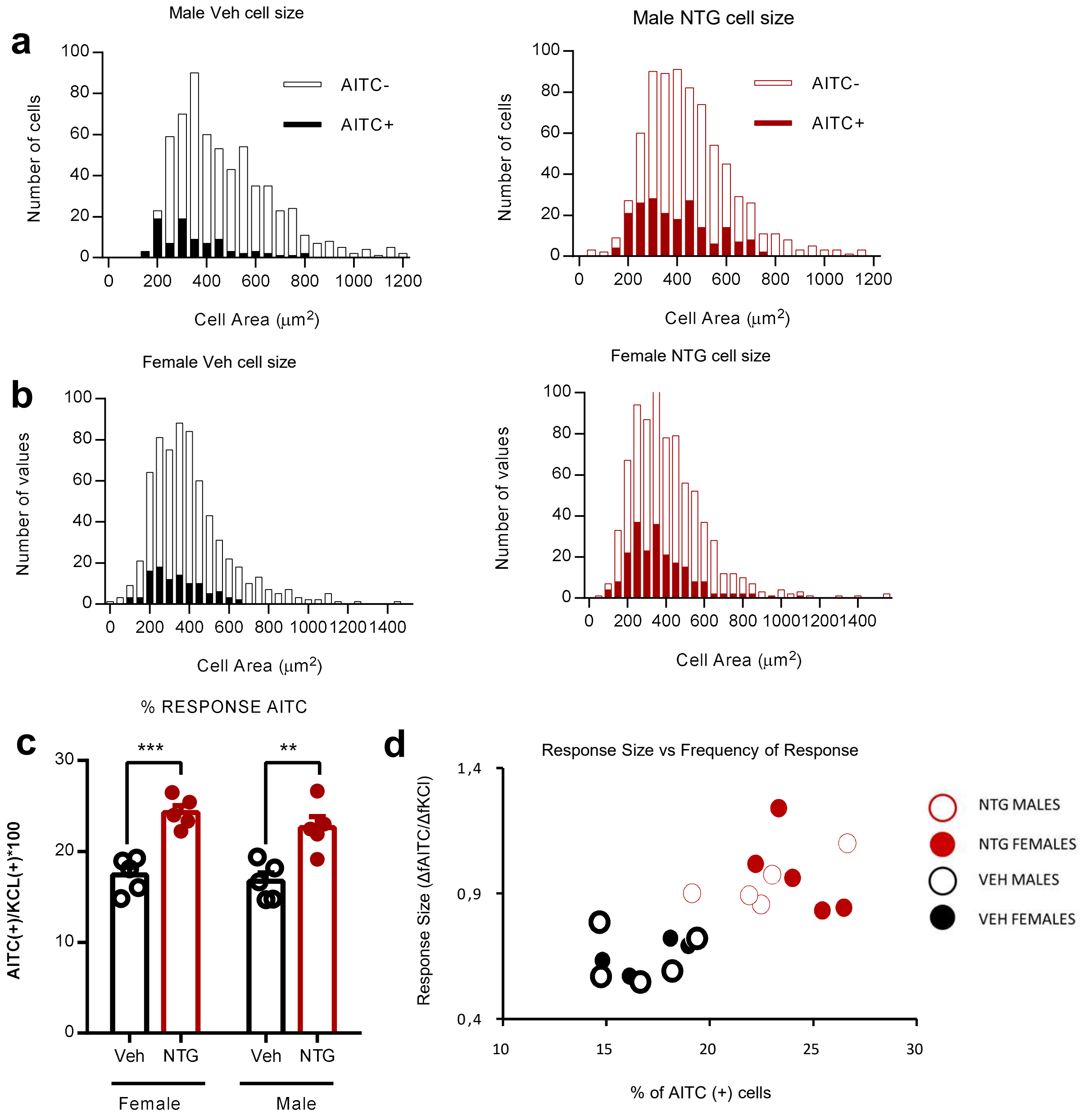

Supplementary Figure 2. AITC activates small to medium-sized neurons in trigeminal cultures, and percentage of AITC-sensitive neurons increases in samples of mice chronically treated with nitroglycerin. a, b Size (μm^2) of neurons responding to AITC (dark) or AITC-unresponsive (light) in samples of vehicle (left, **a**) and nitroglycerin-exposed males (right, **a**) and in samples of vehicle and nitroglycerin-exposed females (**b**). AITC-responding cells form a population of small to medium size neurons. **c** Percentage of AITC-sensitive neurons defined by their sensitivity to KCl is similarly increased in cultures from male and female nitroglycerin-treated mice. ***p0.001, **p<0.01**,** 2-way ANOVA, n=5 cultures from independent animals for each experimental condition, of 3 independent experiments. Mean ± S.E.M. **d** Response size to AITC is proportional to the percentage of AITC-sensitive neurons defined by their sensitivity to KCl. r^2 =0.5545; p<0.001. Linear regression n=5 mice per condition. NTG, nitroglycerin. Veh, Vehicle. Source data are provided in Source Data file, statistical results in Supplementary table 7.

Supplementary Figure 3

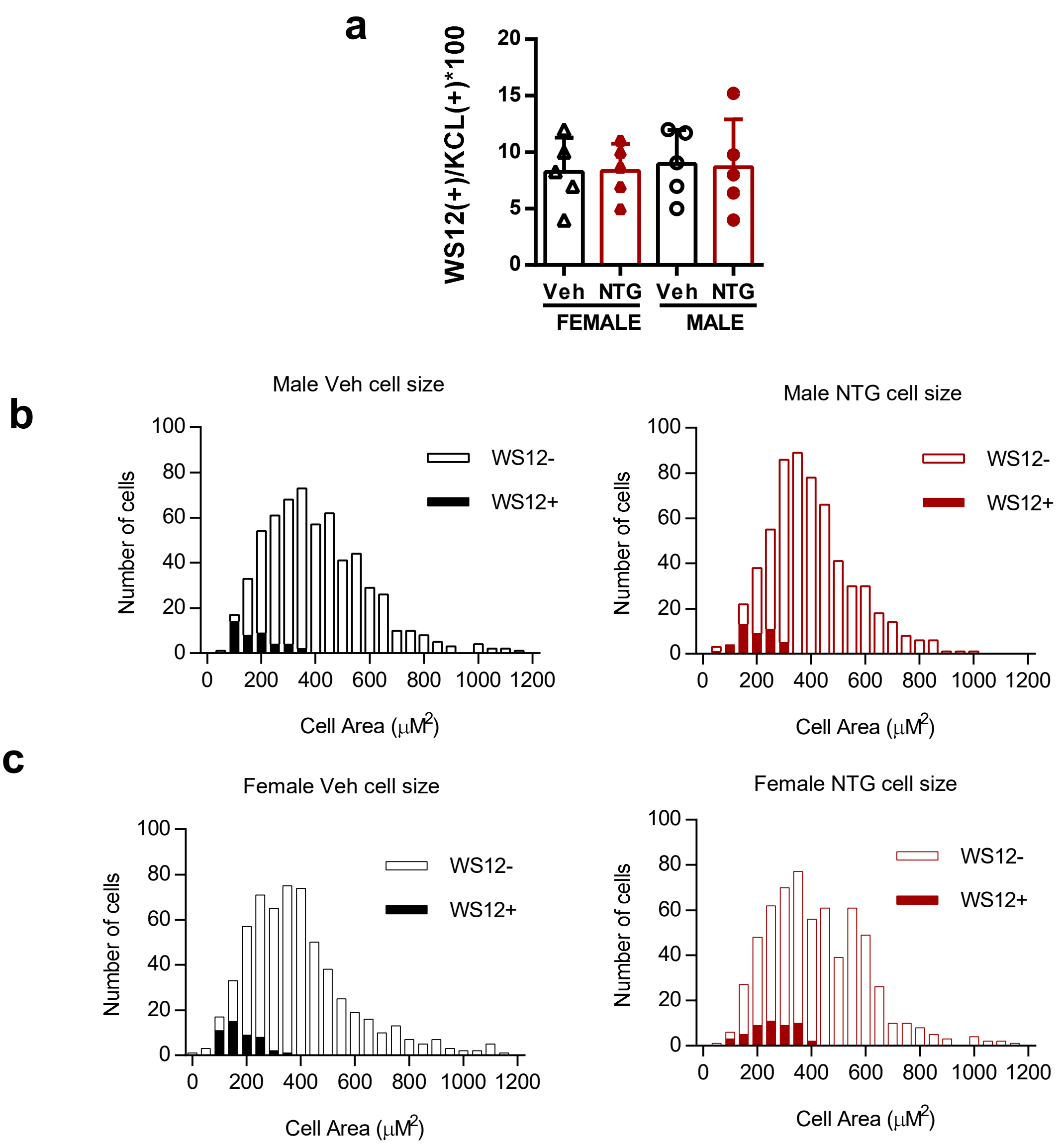

Supplementary Figure 3. Trigeminal TRPM8 expression and responsiveness is similar in male and female mice after chronic nitroglycerin or vehicle. A Percentage of neurons responding to WS12 is similar in males and females exposed to nitroglycerin or vehicle. Mean expression ± S.E.M, datapoints represent independent experiments. 2-way ANOVA n=5 per condition. **B, C** Size (μm^2) of trigeminal neurons responding to WS12 (dark-filled) or WS12-unresponsive (white-filled) in cultures of vehicle (left, **A**) and nitroglycerin-exposed males (right, **A**) and in cultures of vehicle and nitroglycerin-exposed females (**B**). WS12-responding cells form a population of small size neurons. NTG, nitroglycerin. Veh, Vehicle. Source data are provided in Source Data file, statistical results in Supplementary table 8.

Supplementary Figure 4

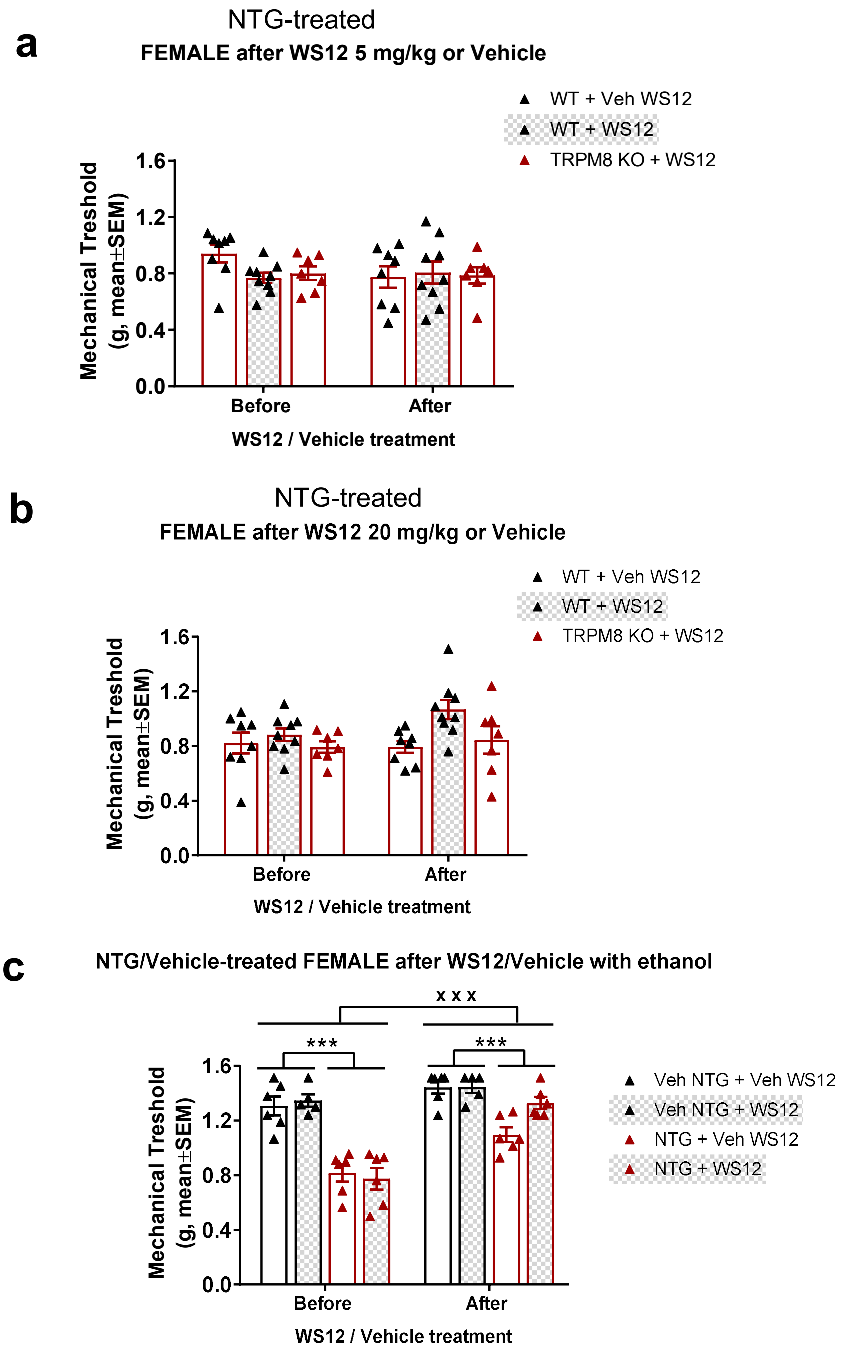

Supplementary Figure 4. Effects of WS12 on mechanical hypersensitivity of nitroglycerin or vehicle-treated female. a WS12 5 mg/kg or its vehicle (2.5% DMSO in corn oil) did not modify mechanical sensitivity of female mice chronically treated with nitroglycerin. **b** WS12 20 mg/kg did not modify mechanical sensitivity of female mice chronically treated with nitroglycerin although a trend (p=0.053) was observed when comparing WS12 and vehicle-treated mice. **c** Administration of WS12 in a vehicle containing 5% ethanol induced significant alleviation in female mice treated with chronic nitroglycerin, although an effect of the vehicle was noted. Mean mechanical thresholds ± S.E.M. (**a**, **b**) 2-way RM ANOVA n=7-9 per group. (**c**) ***p<0.001, 3-way RM ANOVA, n=6-7 per group. NTG, nitroglycerin. Veh, Vehicle. Exact n and source data are available in the Source data file. Source data are provided in Source Data file, statistical results in Supplementary table 9.

Supplementary Figure 5

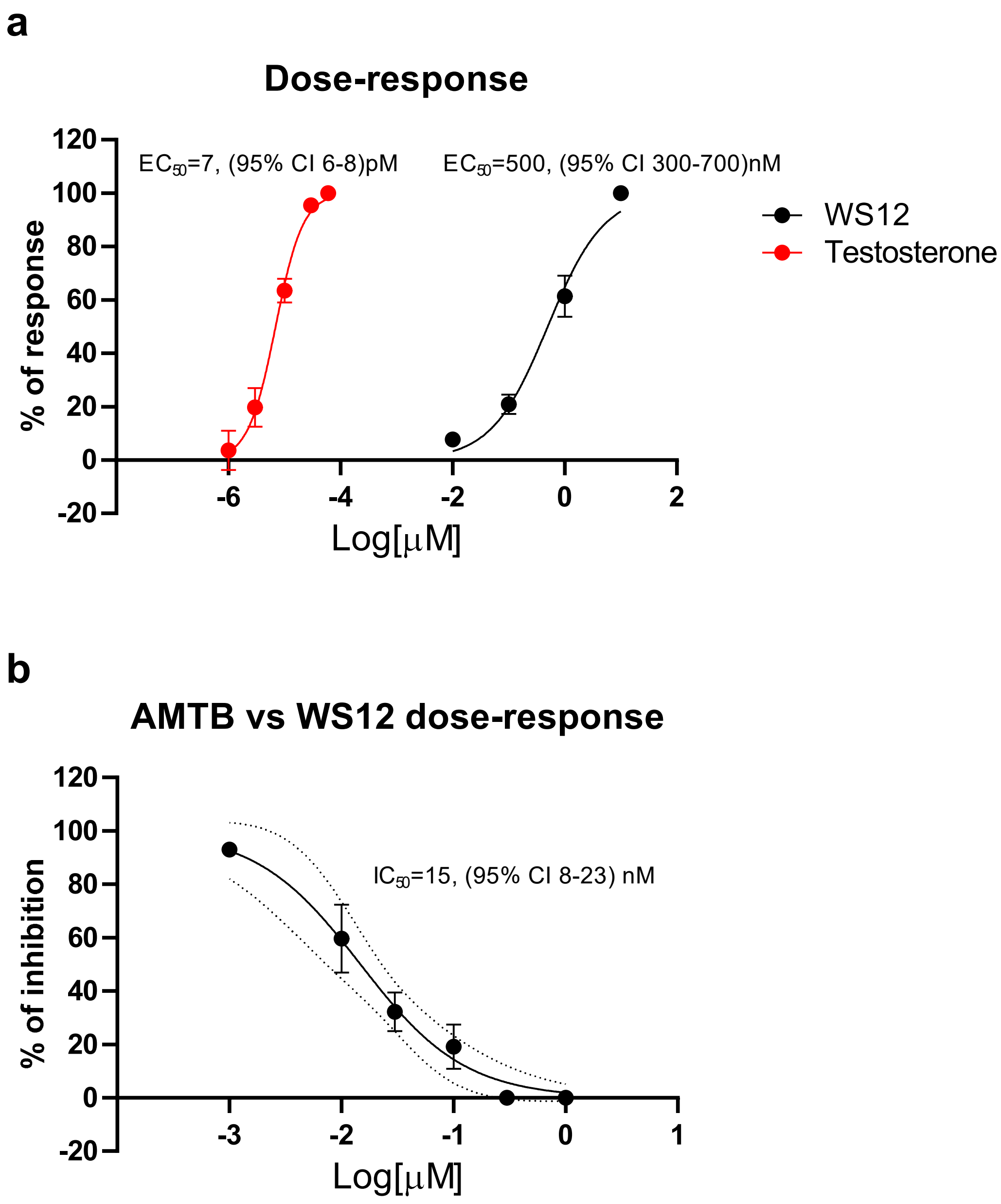

Supplementary Figure 5. Percentage response curves of patch-clamp recordings in HEK293 cells heterologously expressing human TRPM8 after increasing concentrations of testosterone or WS12 and after 500 nM WS12 in the presence of increasing concentrations of AMTB. a Testosterone (EC50=7 pM, n=9 cells from 2 independent cultures) and WS12 (EC50=500 nM, n=5 cells from 2 independent cultures) elicit dose-dependent current responses in HEK293 cells heterologously expressing TRPM8. **b** AMTB (IC50=15 nM, n=8 cells from 2 independent cultures) inhibits dose-dependently the currents induced by 500 nM WS12. Percentage of response was normalized to maximal response. Error bars are SEM, CI: Confidence Interval. n: number of registered cells. EC50s and IC50 were estimated fitting data to a sigmoidal dose-response curve with constraints (Top=100, Bottom=0) using GraphPad Prism Software. Source data are provided in Source Data file, statistical results in Supplementary table 10.

Supplementary Figure 6

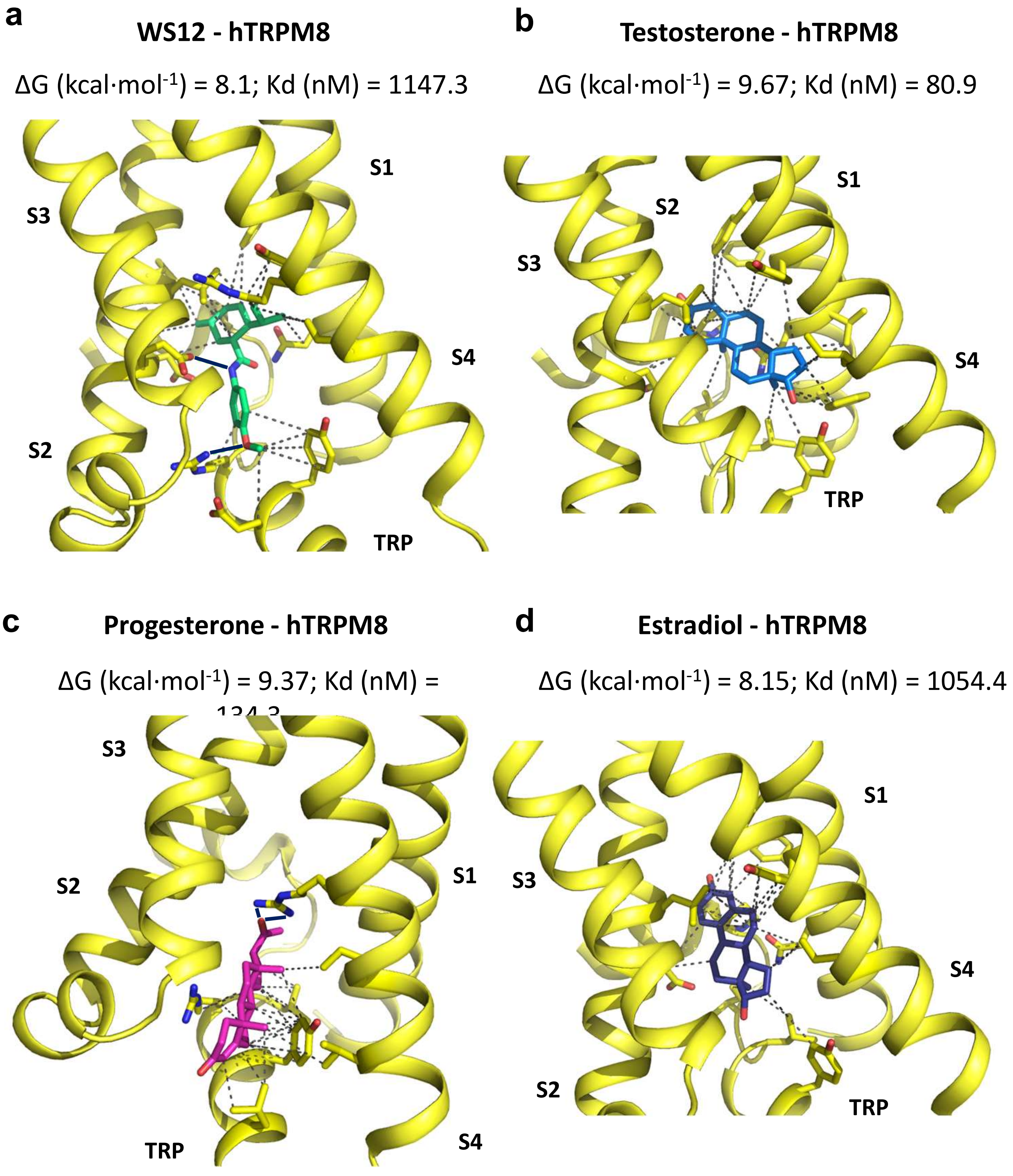

Supplementary Figure 6. Binding of the menthol derivative WS12 and sexual hormones to the human TRPM8 menthol pocket after local docking simulations.
a WS12 **b** Testosterone **c** Progesterone **d** Estradiol. The TRPM8 structure is shown as yellow cartoon representing with sticks the side chains involved in the binding. WS12 and testosterone are shown as sticks of different color. Gray-dashed lines indicate Van der Waals interactions with ligands, whereas blue-solid lines are hydrogen bonds. Hydrogen atoms have been removed. S1, S2, S3, S4 and TRP are TRPM8 transmembrane domains. ΔG is an estimation of the binding energy. Kd is the dissociation constant calculated with ΔG using the equation ΔG=R·T·lnKd. Methods and materials for docking in Supplementary methods within this file and in Supplementary file molecular_docking.zip

Supplementary Methods

S1. Experimental design

S1.1. Characterization of the mechanical sensitization induced by chronic nitroglycerin (NTG) exposure in wild-type male and female mice. (Figure 1 and Supplementary Figures 1A-B). Using the von Frey test, male or female mice were evaluated for basal mechanical sensitivity before repeated NTG (n=6-7) or vehicle (n=6) treatment (day -1). Afterwards, mechanical thresholds were measured again every day of treatment, before and 2 h after each injection (days 0, 2, 4, 6 and 8) and up to 20 days after the beginning of the chronic treatment (days 10, 12, 14, 16, 18 and 20). Animals were sacrificed on day 22 and whenever the vehicle was found innocuous trigeminal ganglia and adrenal glands were collected. Trigeminal ganglia were used to quantify TRPA1/TRM8 mRNA expression (Figures 2C and 3C).

Experimental Groups				
Group	Sex	Genotype	Treatment (i.p.)*	Mice tested
A	Male	WT	Vehicle	6
B			NTG 10 mg/kg	7
C	Female		Vehicle	6
D			NTG 10 mg/kg	6-7

* *Vehicle Fig. 1:*
5% dextrose, 0.105% propylene glycol in water
Vehicle Suppl. Fig. 1:
10% ethanol, 20% propylene glycol in saline

	Behavioural experiment time line																									
Time (day)	-3	-2	-1	0	1	2	3	4	5	6	7	8	9	10	11	12	13	14	15	16	17	18	19	20	21	22
Habituation	■	■	■	■		■		■		■		■		■		■		■		■		■		■		
Von Frey (No treatment on this day)			■											■		■		■		■		■		■		
Von Frey right before treatment				■		■		■		■		■														
Treatment				■		■		■		■		■														
Von Frey 2h after treatment				■		■		■		■		■														
Sacrifice and Sample collection																										■

S1.2. ***Assessment of the role of TRPA1 and TRPM8 in the nociceptive sensitization induced by chronic NTG exposure in mice.*** Wild-type (n=5-6) and TRPA1 knockout (TRPA1 KO, n=6, Figures 2A-B) or TRPM8 knockout mice (TRPM8 KO, n=5, Figures 3A-B) of both sexes were tested for mechanical sensitivity at selected time points before the repeated NTG treatment (day -1), before and 2 h after the NTG treatment (days 0 and 8), and on days 14 and 20.

Experimental Groups TRPA1				
Group	Sex	Genotype	Treatment (i.p.)*	Mice tested
A	Male	WT	NTG 10 mg/kg	6
B		TRPA1KO		6
C	Female	WT		6
D		TRPA1KO		6

* *Vehicle:*
5% dextrose, 0.105% propylene glycol in water

Experimental Groups TRPM8				
Group	Sex	Genotype	Treatment (i.p.)*	Mice tested
A	Male	WT	NTG 10 mg/kg	5
B		TRPM8KO		5
C	Female	WT		5
D		TRPM8KO		5

	Behavioural experiment time line																							
Time (day)	-3	-2	-1	0	1	2	3	4	5	6	7	8	9	10	11	12	13	14	15	16	17	18	19	20
Habituation																								
Von Frey (No treatment on this day)																								
Von Frey right before treatment																								
Treatment																								
Von Frey 2h after treatment																								

S1.3. *Assessment of TRPA1 and TRPM8 functionality in cultures of trigeminal ganglia from mice subjected to the chronic NTG treatment*. *In vitro* Fluo4 calcium imaging was used to assess the effect of the chronic NTG treatment on TRPM8 and TRPA1 function. Animals subjected to the chronic NTG treatment were sacrificed on day 22 and trigeminal ganglia cultures were obtained. Calcium imaging experiments were conducted 16 to 24 h after seeding the neurons. In these experiments, TRPA1 activity was first elicited by AITC (70 μM, 10 s) or TRPM8 activity by WS12 (500 nM, 10 s), and KCl (40 mM, 10 s) was infused 180-200 s later as a positive control to identify neuronal responses (Figures 2D-E and 3C-D). For these experiments, each experimental group was obtained from cultures of 5 animals, with 100-200 neurons registered per animal.

Experimental Groups					
Group	Sex	Genotype	Animal treatment (i.p.)*	Mice treated	Neurons / mouse
A	Male	WT	Vehicle	5	100-200
B			NTG 10 mg/kg	5	100-200
C	Female		Vehicle	5	100-200
D			NTG 10 mg/kg	5	100-200

** Vehicle: 5% dextrose, 0.105% propylene glycol in water*

	Mouse treatment time line																							
Time (day)	0	1	2	3	4	5	6	7	8	9	10	11	12	13	14	15	16	17	18	19	20	21	22	23
Animal treatment																								
Sacrifice																								

	Calcium Imaging Assay Time line											
Time (Sec)	0	10	20	...	90	100	110	...	290	300	310	...
AITC 70 μM												
WS12 500 nM												
KCl 40 mM												

AITC vehicle: 0.01% DMSO in HBSS
WS12 vehicle 0.01% DMSO in HBSS
KCl vehicle: HBSS (Extracellular solution)

S1.4. *Assessment of NTG activity on neuronal murine TRPA1 through calcium imaging.* In vitro Fluo4 calcium imaging was used to study NTG-TRPA1 activity in primary trigeminal cultures obtained from untreated wild type and TRPA1 KO mice. In trigeminal ganglia experiments (Figure 2F-G), cells were seeded and 16 to 24 h later were challenged first with NTG (100 μM, 10 s) and subsequently with TRPA1 agonist AITC (70 μM, 10 s). KCl (40 mM, 10 s) was used afterwards to identify and normalize neuronal responses, and a total of 4 animals were used for each group in 4 independent experiments, with 100-200 neurons registered per animal.

Experimental Groups				
Group	Sex	Genotype	Mice	Neurons / mouse
A	Male	WT	4	100-200
B		TRPA1KO	4	100-200

	Calcium Imaging Assay Time line															
Time (Sec)	0	10	20	...	90	100	110	...	390	400	410	...	690	700	710	...
NTG 100 µM																
AITC 70 µM																
KCl 40 mM																

NTG vehicle: 0.115% dextrose + 0.002% propylene glycol in HBSS
AITC vehicle: 0.01% DMSO in HBSS
KCl vehicle: HBSS (Extracellular solution)

S1.5. *Assessment of NTG activity on human TRPA1 through calcium imaging.* The activation of TRPA1 by NTG was also assessed in naïve HEK293 cells, in HEK293 cells transfected with human TRPA1 and in IMR90 cells natively expressing human TRPA1 (Figure 2H). 4 independent experiments were performed, with a total of 200-300 cells analysed per cell type and dose. In each experiment, different sets of HEK293-TRPA1 and IMR90 cells were exposed to NTG concentrations (10, 50 or 100 µM, 10 s) and HEK293 naïve control cells were exposed to 50 µM NTG (10 s). Ionomycin (10 µM, 10 s) was applied at the end to verify calcium transients and to normalize the responses.

Experimental Groups				
Group	Sex	Cell type	Number of experiments	Cells/experiment
A	Female	HEK 293 WT	4 (50 µM NTG)	60-100
B	Female	HEK 293 - TRPA1	4 (for each concentration)	60-100
C	Female	IMR90	4 (for each concentration)	60-100

	Calcium Imaging Assay Time line											
Time (Sec)	0	10	20	...	60	70	80	...	290	300	310	...
NTG 10 µM												
NTG 50 µM												
NTG 100 µM												
Ionomycin 10 µM												

NTG vehicle: 0.115% dextrose + 0.002% propylene glycol in HBSS
Ionomycin vehicle: Water in HBSS

S1.6. *NTG effect on murine CGRP release.* NTG-induced CGRP release was assessed through immunocytochemistry in trigeminal primary cultures subjected to 1 of 3 different treatments: exocytosis inhibitor DD04107 (10 µM, 60 min) followed by NTG (100 µM, 30 min), vehicle of exocytosis inhibitor (saline) followed by NTG, or vehicle of exocytosis inhibitor followed by vehicle of NTG (Figure 2I-J). After 30 min of exposure to the last treatment, cells were processed for immunocytochemistry and CGRP levels inside MAP2-labeled cells were estimated. Each experimental group was obtained by taking 5 representative images from each of 5 cultures obtained from independent animals.

Group	First incubation (60 min)	Second incubation (30 min)
1.Basal condition	DD04107 vehicle	NTG vehicle
2.CGRP release	DD04107 vehicle	NTG
3. Inhibition of CGRP release	DD04107	NTG + DD04107

S1.7. ***Evaluation of the pain-relieving efficacy of TRPM8 agonism in NTG-sensitized female mice***. In these experiments, females were exposed to the chronic treatment with vehicle or NTG and 12 days later (day 20 after beginning of the treatment) were evaluated for mechanical sensitivity. After this measurement, mice were treated with the potent TRPM8 agonist WS12 (10 mg/kg i.p.) or its vehicle to obtain 4 experimental groups: NTG vehicle-WS12 vehicle (n=6), NTG- WS12 vehicle (n=5), NTG vehicle-WS12 (n=6) and NTG-WS12 (n=7, Figure 3F and Supplementary figure 5A). Mechanical sensitivity was assessed again 30 min later.

To assess possible antinociceptive effects of lower and higher WS12 doses, and to confirm TRPM8 participation on these effects, wild-type and TRPM8 KO female mice were chronically treated with NTG and 12 days later, mechanical sensitivity was assessed. Afterwards, mice received acute treatments with WS12 (5 and 20 mg/kg) or its vehicle, and mechanical sensitivity was measured 30 min later. The following NTG-treated groups were obtained: wild-type-WS12 vehicle (n=8), wild-type-WS12 (n=9) and TRPM8 KO-WS12 (n=7, Supplementary figures 5B-5C).

Experimental Groups					
Group	Sex	Genotype	Treatment 1 (i.p.)*[1]	Treatment 2 (i.p.)*[2]	Mice tested
A	Female	WT	Vehicle	Vehicle	6
B			NTG 10 mg/kg		7
C			Vehicle	WS12 10 mg/kg	6
D			NTG 10 mg/kg		6-7

*[1] *Vehicle: 5% dextrose, 0.105% propylene glycol in water*

*[2] *Vehicle: 5% ethanol in 45% 2-Hydroxypropyl-β-cyclodextrin dissolved in water,* ***or*** *2.5% DMSO in corn oil*

Experimental Groups					
Group	Sex	Genotype	Treatment 1 (i.p.)*[1]	Treatment 2 (i.p.)*[2]	Mice tested
A	Female	WT	NTG 10 mg/kg	Vehicle	8
B				WS12 20 mg/kg	9
C		TRPM8KO			7

*[1] *Vehicle: 5% dextrose, 0.105% propylene glycol in water*

*[2] *Vehicle: 2.5% DMSO in corn oil*

	Time line behavioural experiment																							
Time (day)	-3	-2	-1	0	1	2	3	4	5	6	7	8	9	10	11	12	13	14	15	16	17	18	19	20
Habituation				■								■						■					■	■
Treatment 1				■		■		■		■		■												
Von Frey right before treatment 2																								■
Treatment 2																								■
Von Frey 30 min after treatment 2																								■

S1.8. *Evaluation of the pain-relieving efficacy of TRPM8 agonism in formalin-sensitized female mice*. To rule out possible antinociceptive effects of WS12 in females subjected to TRPA1-mediated pain, the effect of WS12 was evaluated after local administration (i.pl.) in the formalin test, a TRPA1-dependent model of acute pain. Formalin was co-injected with 6 nmol of WS12 (n=4) or its vehicle (n=7) in the right hind paw of wild-type females, and licking behaviour was quantified for 1 h (Figure 3F).

Experimental Groups				
**Group**	**Sex**	**Genotype**	**Treatment (i.pl.)***	**Mice tested**
A	**Female**	**WT**	**5% Formalin in vehicle**	**7**
B			**5% Formalin in 6 nmol WS12 in vehicle**	**4**

** Vehicle: 0.6% DMSO in 45% 2-Hydroxypropyl-β-cyclodextrin in water*

	Time line behavioural experiment												
**Time (min)**	**0**	**5**	**10**	**15**	**20**	**25**	**30**	**35**	**40**	**45**	**50**	**55**	**60**
**Treatment 1**													
**Quantification of licking behaviour**													

S1.9. *Evaluation of endogenous protective TRPM8 activity in NTG-exposed male mice*. Further experiments were designed to discriminate a possible endogenous protective activity of TRPM8 in wild-type males recovered from NTG-induced pain (Figures 3G-I). Male mice recovered from NTG-induced hypersensitivity (n=6, 12 days after ending NTG injections) were treated every other day with increasing doses of the specific TRPM8 blocker AMTB (5, 10 or 15 mg/kg) or its vehicle, and mechanical sensitivity was assessed 30 min after each treatment (Figure 3G).

Single Experimental Group (Within design)								
**Group**	**Sex**	**Genotype**	**Treatment 1 (i.p.)*1**	**Treatment 2 (i.p.)*2**	**Treatment 3 (i.p.)*2**	**Treatment 4 (i.p.)*2**	**Treatment 5 (i.p.)*2**	**Mice tested**
A	**Male**	**WT**	**NTG 10 mg/kg**	**AMTB Vehicle**	**AMTB 5 mg/kg**	**AMTB 10 mg/kg**	**AMTB 15 mg/kg**	**6**

**1 Vehicle: 5% dextrose, 0.105% propylene glycol in water*

**2 Vehicle: 2.5% DMSO in saline*

	Time line behavioural experiment																												
**Time (day)**	**0**	**1**	**2**	**3**	**4**	**5**	**6**	**7**	**8**	**9**	**10**	**11**	**12**	**13**	**14**	**15**	**16**	**17**	**18**	**19**	**20**	**21**	**22**	**23**	**24**	**25**	**26**	**27**	**28**
**Habituation**																													
**Treatment 1**																													
**Treatment 2 with AMTB Vehicle**																													
**Treatment 3 AMTB 5 mg/kg**																													
**Treatment 4 AMTB 10 mg/kg**																													
**Treatment 5 AMTB 15 mg/kg**																													
**Von Frey**																													

S1.10. *Evaluation of endogenous protective TRPM8 activity in formalin-exposed male mice*. An additional experiment was conducted to investigate the presence of endogenous protective TRPM8 tone after formalin-induced pain in males (Figure 3H). In this case, males were exposed to 5% i.pl. formalin and licking behaviour was quantified for 1 h until cessation of nocifensive behaviour. Then, mice received AMTB 10 mg/kg i.p. (n=6) or its vehicle (n=6) and quantification of licking behaviour continued for 1 h.

Experimental Groups					
Group	Sex	Genotype	Treatment 1 (i.pl.)*[1]	Treatment 2 (i.p.)*[2]	Mice tested
A	Male	WT	5% Formalin	Vehicle	6
B				AMTB 10 mg/kg	6

*[1] *Vehicle: Saline*

*[2] *Vehicle: 2.5% DMSO in saline*

	Time line behavioural experiment																								
Time (min)	0	5	10	15	20	25	30	35	40	45	50	55	60	65	70	75	80	85	90	95	100	105	110	115	120
Treatment 1																									
Quantification of licking behaviour																									
Treatment 2																									

S1.11. *Assessment of the role of testicles on the progress of mechanical sensitivity in male mice exposed to the model of chronic migraine.* (Figure 4A) Baseline mechanical sensitivity was measured and mice were subjected to orchidectomy or to a sham surgery. After 3 weeks, sham and orchidectomized males were exposed to the chronic treatment with NTG 10 mg/kg or its vehicle (i.p., days 0, 2, 4, 6, 8). 4 experimental groups were obtained: sham-vehicle (n=6), sham-NTG (n=6), orchidectomized-vehicle (n=5) and ochidectomized-NTG (n=7). Mechanical sensitivity was measured again before and 2 h after the NTG injections on days 0 and 8, and on days 14 and 20.

Experimental Groups					
Group	Sex	Genotype	Surgery	Treatment (i.p.)*	Mice tested
A	Male	WT	Sham	Vehicle	6
B				NTG 10 mg/kg	6
C			Orchidectomy	Vehicle	5
D				NTG 10 mg/kg	7

* *Vehicle: 5% dextrose, 0.105% propylene glycol in water*

	Time line behavioural experiment																										
Time (day)	-17	-16	-15	-14	...	...	0	1	2	3	4	5	6	7	8	9	10	11	12	13	14	15	16	17	18	19	20
Habituation																											
Surgery																											
Von Frey (No treatment on this day)																											
Von Frey right before treatment																											
Treatment																											
Von Frey 2h after treatment																											

S1.12. *Elucidation of testosterone and TRPM8 involvement on the recovery of normal sensitivity observed in male mice after cessation of the chronic NTG treatment.* To determine whether testosterone could provide mechanical pain relief, and to discriminate the role of TRPM8 in this pain relief (Figure 4B), wild-type and TRPM8 KO male mice were measured for baseline mechanical sensitivity and all mice were orchidectomized. 3 weeks later, mechanical sensitivity was again assessed and mice were implanted with alzet osmotic minipumps (Model 2004, Durect Corporation, Cupertino, CA, USA) filled with testosterone or its vehicle. 4 experimental groups with orchidectomized males were obtained: wild-type mice receiving vehicle (n=5) or testosterone infusions (n=6, 6 µg/h) and TRPM8 KO mice receiving vehicle (n=6) or testosterone (n=7). 3 days later, mechanical sensitivity was assessed and males were exposed to

the chronic treatment with NTG 10 mg/kg or its vehicle (i.p., days 0, 2, 4, 6, 8). Mechanical sensitivity was measured (before and 2 h after NTG injections on days 0 and 8, and on days 14 and 20). To further discriminate the involvement of TRPM8 on the pain-relieving effects of exogenous testosterone, all mice were exposed to a dose of AMTB (10 mg/kg i.p.) on day 20 and mechanical sensitivity was evaluated again 30 min later (Figure 4C).

Experimental Groups							
Group	Sex	Genotype	Surgery	Treatment 1 (Minipump)*1	Treatment 2 (i.p.)*2	Treatment 3 (i.p.)*3	Mice tested
A	Male	WT	Orchidectomy	Vehicle	NTG 10 mg/kg	AMTB 10 mg/kg	5
B				Testosterone 6 µg /h			6
C		TRPM8KO		Vehicle			6
D				Testosterone 6 µg /h			7

*1 *Vehicle: 45% 2-Hydroxypropyl-β-cyclodextrin in water*

*2 *Vehicle: 5% dextrose, 0.105% propylene glycol in water*

*3 *Vehicle: 2.5% DMSO in saline*

	Time line behavioural experiment																									
Time (day)	-17	-16	-15	-14	...	-4	-3	...	0	1	2	3	4	5	6	7	8	9	...	13	14	15	...	19	20
Habituation																									
Surgery																									
Minipump implantation																									
Von Frey (No treatment on this day)																									
Treatment 1																									
Von Frey right before treatment																									
Treatment 2																									
Von Frey 2h after treatment 2																									
Treatment 3																									
Von Frey 30 min after treatment 3																									

S1.13. *Elucidation of the antinociceptive effect of testosterone-TRPM8 activity in female mice exposed to chronic NTG.* Finally, one last behavioural experiment was conducted to discriminate possible TRPM8-mediated effects of exogenous testosterone on NTG-sensitized female mice (Figure 4D). Wild-type and TPRM8 KO females receiving NTG were evaluated for mechanical sensitivity after 12 days of ending the chronic treatment. Then, females received a single i.p. dose of testosterone 10 mg/kg or its vehicle, obtaining 3 groups: wild-type-testosterone (n=9), wild-type-testosterone vehicle (n=8) and TRPM8 KO-testosterone (n=7). Mechanical sensitivity was measured again 1 h later.

Experimental Groups					
Group	Sex	Genotype	Treatment 1 (i.p.)*1	Treatment 2 (i.p.)*2	Mice tested
A	Female	WT	NTG 10 mg/kg	Vehicle	8
B				Testosterone 1 mg/kg	9
C		TRPM8KO			7

*1 *Vehicle: 5% dextrose, 0.105% propylene glycol in water*

*2 *Vehicle: 2.5% DMSO in corn oil*

	Time line behavioural experiment																							
Time (day)	-3	-2	-1	0	1	2	3	4	5	6	7	8	9	10	11	12	13	14	15	16	17	18	19	20
Habituation																								
Treatment 1																								
Von Frey right before treatment 2																								
Treatment 2																								
Von Frey 1 h after treatment 2																								

S1.14. *Assessment of testosterone activity on neuronal TRPM8 through calcium imaging.* Trigeminal cultures from naïve wild-type and TRPM8 KO mice were exposed to testosterone and subsequently to TRPM8 agonist WS12 (Figure 4E), followed by KCl for neuronal identification and normalization. A total of 4 animals were used for each experimental group (wild-type or TRPM8 KO) in 4 independent experiments, with 100-200 neurons registered per animal.

Experimental Groups				
Group	Sex	Genotype	Mice	Neurons / mouse
A	Male	WT	4	100-200
B		TRPM8KO	4	100-200

	Calcium Imaging Assay Time line															
Time (Sec)	0	10	20	...	90	100	110	...	390	400	410	...	690	700	710	...
Testosterone 10 pM						■										
WS12 500 nM										■						
KCl 40 mM														■		

Testosterone vehicle: 0.01% DMSO in HBSS (Extracellular solution)
WS12 vehicle 0.01% DMSO in HBSS
KCl vehicle: HBSS

S1.15. *Assessment of testosterone activity on human and rat TRPM8 through calcium imaging.* Cultures of HEK293 naïve cells, HEK293 cells expressing human TRPM8 or HEK293 cells expressing rat TRPM8 were exposed to testosterone and to ionomycin to verify calcium transients and for normalization (Figure 4F). 3 independent experiments were conducted with a total of 200-300 cells per each cell type.

Experimental Groups				
Group	Sex	Cell type	Number of experiments	Cells/experiment
A	Female	HEK 293 WT	3	60-100
B		HEK 293 - hTRPM8	3	60-100
C		HEK 293 - rTRPM8	3	60-100

	Calcium Imaging Assay Time line											
Time (Sec)	0	10	20	...	50	60	70	...	390	40	410	...
Testosterone 10 pM						■						
Ionomicin 10 µM											■	

WS12 vehicle: 0.01% DMSO in HBSS (Extracellular solution)
Ionomicin vehicle: Water in HBSS

S1.16. ***Evaluation of testosterone activity on human TRPM8 through patch-clamp electrophysiology.*** HEK293 cells expressing human TRPM8 were exposed to a voltage ramp from -120 to +120 mV of 300 ms duration, under basal conditions (n=5) or after 30s 1 pM testosterone pulses with (n=5) or without (n=7) the presence of 10 μM AMTB.

Experimental Groups					
Group	Sex	Cell type	Treatment 1*	Treatment 2*	Cells
A	Female	HEK 293 - hTRPM8	*Untreated*		5
B			Vehicle	Testosterone 1 pM	7
C			ATMB 10 μM		5

**Vehicle: 0.1% DMSO in extracellular solution*

	Patch clamp assay time line										
Time (Sec)	0	10	20	30	40	50	60	70	80	90	100
Treatment 1											
Treatment 2											

S1.16. ***Impact of androgen receptor expression on human TRPM8 electrophysiological activity.*** HEK293 cells expressing human TRPM8 were first transfected with a plasmid coding for GFP together with a plasmid coding for either siRNA designed against the androgen receptor (AR) or scrambled siRNA. In parallel, naïve HEK293 cells lacking TRPM8 were exposed to the same culture conditions without transfection. After 72 hours, hTRPM8-AR siRNA cells (n=7), hTRPM8-Scrambled siRNA (n=6) and naïve HEK293 (n=6) cells were exposed to a voltage ramp from -120 to +120 mV of 300 ms duration after 30s 1 pM testosterone pulses. Replicates for the three different groups/cultures (hTRPM8-AR siRNA, hTRPM8-Scrambled siRNA, naïve HEK293) treated in parallel were used to assess AR expression through western blot.

Experimental Groups					
Group	Sex	Cell type	Treatment 1*[1]	Treatment 2*[2]	Cells
A	Female	HEK 293 - hTRPM8	GFP - Androgen Receptor siRNA	Testosterone 1 pM	7
B			GFP - Scrambled siRNA		6
C		HEK 293	*Untreated*		6

*[1] *Vehicle: DEPC-treated water*

*[2] *Vehicle: 0.1% DMSO in HBSS (extracellular solution)*

	Transfection time line				*Patch clamp assay time line*										
Time (days)	1	2	3	Time (Sec)	0	10	20	30	40	50	60	70	80	90	100
Treatment 1															
Treatment 2															

S2. Computational modelling. A homology model of human TRPM8 channel was designed considering the structure of the TRPM8 from *Ficedula albicollis*, determined by cryo-electron microscopy at 4.1 Å (Protein Data Bank code 6BPQ, https://www.rcsb.org/structure/6BPQ). The sequence of human TRPM8 (Uniprot Q7Z2W7, https://www.uniprot.org/uniprotkb/Q7Z2W7/entry) was modeled against the reference structure, following the standard protocol implemented by Yasara (version 21.12.19, http://www.yasara.org/). Sequence alignments

between human TRPM8 and *F. albicollis* were performed with ClustalO (1.2.4) from the European Bioinformatic Institute (EBI, https://www.ebi.ac.uk/).
Blind docking experiments were carried out with AutoDock implemented in Yasara. WS12 (PubChem CID: 11266244, https://pubchem.ncbi.nlm.nih.gov/compound/11266244) and testosterone (PubChem CID: 6013, https://pubchem.ncbi.nlm.nih.gov/compound/6013) structures were obtained from the National Center for Biotechnology Information (NCBI) PubChem database. 800 docking runs with flexible ligands were fixed and results clustered around binding hot spots. By using the Assisted Model Building with Energy Refinement (AMBER03) force field, a simulated annealing optimization of the complexes was performed, which moved the structure to a stable energy minimum. The best binding energy in each cluster was saved and solutions grouped according to putative TRPM8 binding sites.
Local docking experiments focused in the menthol binding pocket were also performed with progesterone (PubChem CID: 5994, https://pubchem.ncbi.nlm.nih.gov/compound/5994) and estradiol (PubChem CID: 5757, https://pubchem.ncbi.nlm.nih.gov/compound/5757). A total of 50 runs were set with the side chain of critical residues in the menthol binding pocket kept flexible. Figures were drawn with open source PyMol (The PyMol Molecular Graphics System, version 2.5.0 Schrödinger, LLC, https://pymol.org/2/).

Supplementary Statistical Results

Supplementary table 1. Statistical results for Figure 1.

	Statistical analysis		Result
**(b) Mechanical sensitivity 2h after nitroglycerin or vehicle treatments in males**	2-way RM ANOVA	Day<br>Nitroglycerin<br>Nitroglycerin*day	F(4,44)=2.370; p=0.067<br>F(1,11)=119.145; p=0.000<br>F(4,44)=2.674; p=0.044
**(b) Mechanical sensitivity before and after chronic nitroglycerin or vehicle in males**	2-way RM ANOVA	Day<br>Nitroglycerin<br>Nitroglycerin*day	F(10,110)=12.090; p=0.000<br>F(1,11)=77.427; p=0.000<br>F(10,110)=9.247; p=0.000
**(b) Mechanical sensitivity 2h after nitroglycerin or vehicle treatments in females**	2-way RM ANOVA	Day<br>Nitroglycerin<br>Nitroglycerin*day	F(4,44)=2.036; p=0.106<br>F(1,11)=103.651; p=0.000<br>F(4,44)=1.094; p=0.371
**(b) Mechanical sensitivity before and after chronic nitroglycerin or vehicle in females**	2-way RM ANOVA	Day<br>Nitroglycerin<br>Nitroglycerin*day	F(10,110)=4.344; p=0.000<br>F(1,11)=77.730; p=0.000<br>F(10,110)=4.249; p=0.000

RM ANOVA, Repeated Measures Analysis of Variance; F, F-test.

Supplementary table 2. Statistical results for Figure 2.

	Statistical analysis		Result
**(a) Mechanical sensitivity 2h after nitroglycerin treatments in TRPA1KO or wild-type males**	2-way RM ANOVA	Day<br>Genotype<br>Genotype*day	F(1,10)=1.146; p=0.309<br>F(1,10)=28.694; p=0.000<br>F(1,10)=0.252; p=0.626
**(a) Mechanical sensitivity before and after chronic nitroglycerin in TRPA1KO or wild-type males**	2-way RM ANOVA	Day	F(3,30)=12.272; p=0.000
**(a) Mechanical sensitivity 2h after nitroglycerin treatments in TRPA1KO or wild-type females**	2-way RM ANOVA	Day<br>Genotype<br>Genotype*day	F(1,9)=0.000; p=0.998<br>F(1,9)=103.651; p=0.001<br>F(1,9)=0.186; p=0.676
**(a) Mechanical sensitivity before and after chronic nitroglycerin in TRPA1KO or wild-type females**	2-way RM ANOVA	Day	F(3,27)=13.849; p=0.000
**(b) TRPA1 mRNA levels from trigeminal ganglia in wild-type animals treated with vehicle or nitroglycerin**	2-way ANOVA	Genotype	F(1,3)=22.525; p=0.001
**(d) Size of calcium responses to AITC in trigeminal cultures from mice treated with vehicle or nitrolgycerin**	2-way ANOVA	Genotype*day	F(3,27)=5.717; p=0.004
**(f) Percentage of trigeminal neurons from wild-type and TRPA1KO mice responding to nitroglycerin and AITC**	Mann-Whitney U	Genotype	p=0.0286
**(i) αCGRP immunoreactivity of trigeminal cultures after vehicle, nitroglycerin or nitroglycerin+DD04107**	One-way ANOVA	Treatment	F(4, 122); p=0.0058

TRPA1, Transient receptor potential Ankyrin 1; ANOVA, Analysis of Variance; AITC, Allyl Isothiocyanate; TRPA1KO, TRPA1 knockout; RM, Repeated Measures; F, F-test.

Supplementary table 3. Statistical results for Figure 3.

	Statistical analysis	Result	
**(a) Mechanical sensitivity 2h after nitroglycerin treatments in TRPM8KO or wild-type males**	2-way RM ANOVA	Day<br>Genotype<br>Genotype*day	F(1,8)=0.882; p=0.375<br>F(1,8)=0.000; p=0.991<br>F(1,8)=0.407; p=0.541
**(a) Mechanical sensitivity before and after chronic nitroglycerin in TRPM8KO or wild-type males**	2-way RM ANOVA	Day<br>Genotype<br>Genotype*day	F(3,24)=17.941; p=0.000<br>F(1,8)=5.658; p=0.045<br>F(3,24)=3.204; p=0.041
**(a) Mechanical sensitivity 2h after nitroglycerin treatments in TRPM8KO or wild-type females**	2-way RM ANOVA	Day<br>Genotype<br>Genotype*day	F(1,8)=0.155; p=0.704<br>F(1,8)=2.867; p=0.129<br>F(1,8)=0.000; p=0.985
**(a) Mechanical sensitivity before and after chronic nitroglycerin in TRPM8KO or wild-type females**	2-way RM ANOVA	Day<br>Genotype<br>Genotype*day	F(3,24)=28.839; p=0.000<br>F(1,8)=0.529; p=0.488<br>F(3,24)=0.870; p=0.470
**(b) TRPM8 mRNA levels from trigeminal ganglia in wild-type animals treated with vehicle or nitroglycerin**	2-way ANOVA	Sex<br>Treatment<br>Sex*Treatment	F(1, 20)=0.169; p=0.685<br>F(1, 20)= 0.001; p=0.976<br>F (1, 20)=0.257; p = 0.574
**(d) Size of calcium responses to WS12 of trigeminal cultures from male and female mice treated with vehicle or nitroglycerin**	2-way ANOVA	Sex<br>Treatment<br>Sex*Treatment	F(1, 16)=0.209; p= 0.654<br>F(1, 16)=0.582; p= 0.457<br>F(1, 16)=0.002; p= 0.962
**(e) Mechanical sensitivity in vehicle or nitroglycerin-treated wild-type females before and after WS12 or its vehicle**	3-way RM ANOVA	Day<br>Day*WS12<br>Day*Nitroglycerin<br>Day*WS12*Nitroglycerin<br>WS12<br>Nitroglycerin<br>WS12*Nitroglycerin	F(1,20)=2.524; p=0.128<br>F(1,20)=0.166; p=0.688<br>F(1,20)=5.213; p=0.033<br>F(1,20)=1.451; p=0.242<br>F(1,20)=0.056; p=0.816<br>F(1,20)=32.065; p=0.000<br>F(1,20)=2.377; p=0.139
**(f) Formalin-induced nocifensive behavior in female mice receiving local WS-12 or vehicle**	Unadjusted Repeated T-tests	0-5 min<br>10-60 min	p=0.019<br>p>0.123 for all time intervals
**(g) Mechanical sensitivity after AMTB doses or vehicle in nitroglycerin pre-exposed wild-type males**	Friedman Test		P=0.0206
**(h) Nocifensive behavior in male mice treated with formalin and receiving posterior ATMB or vehicle**	Unadjusted Repeated T-tests	0-60 min after formalin<br>30-35 min after AMTB<br>0-30, 35-60 after AMTB	p>0.146<br>p=0.036<br>p>0.102

TRPM8, Transient Receptor Potential Melastatin 8; TRPM8KO, TRPM8 knockout; RM, Repeated Measures; ANOVA, Analysis of Variance; WS12, TRPM8 agonist (1R,2S,5R)-2-Isopropyl-N-(4-methoxyphenyl)-5-methylcyclohexanecarboxamide; AMTB, TRPM8 blocker, N-(3-aminopropyl)-2-{[(3-methylphenyl) methyl]oxy}-N-(2-thienylmethyl)benzamide hydrochloride; F, F-test.

Supplementary table 4. Statistical results for Figure 4.

	Statistical analysis	Result	
**(a) Mechanical sensitivity 2h after vehicle or nitroglycerin treatments in sham-operated or orchidectomized animals**	3-way RM ANOVA	Day<br>Day*Surgery<br>Day*Nitroglycerin<br>Day*Surgery*Nitroglycerin<br>Surgery<br>Nitroglycerin<br>Surgery*Nitroglycerin	F(1,20)=0.304; p=0.587<br>F(1,20)=0.012; p=0.572<br>F(1,20)=0.052; p=0.242<br>F(1,20)=0.013; p=0.557<br>F(1,20)=0.122; p=0.730<br>F(1,20)=79.340; p=0.000<br>F(1,20)=1.702; p=0.207

**(a) Mechanical sensitivity before and after chronic nitroglycerin or vehicle in sham-operated or orchidectomized animals**	3-way RM ANOVA	Day<br>Day*Surgery<br>Day*Nitroglycerin<br>Day*Surgery*Nitroglycerin<br>Surgery<br>Nitroglycerin<br>Surgery*Nitroglycerin	F(4,80)=30.870; p=0.000<br>F(4,80)=1.364; p=0.254<br>F(4,80)=12.044; p=0.000<br>F(4,80)=2.035; p=0.097<br>F(1,20)=2.360; p=0.140<br>F(1,20)=38.960; p=0.000<br>F(1,20)=6.957; p=0.016
**(b) Mechanical sensitivity 2h after nitroglycerin in orchidectomized wild-type or TRPM8 knockout mice supplemented with testosterone or vehicle**	3-way RM ANOVA	Day<br>Day*Genotype<br>Day*Nitroglycerin<br>Day*Genotype*Nitroglycerin<br>Genotype<br>Nitroglycerin<br>Genotype*Nitroglycerin	F(1,20)=2.179; p=0.155<br>F(1,20)=10.146; p=0.005<br>F(1,20)=0.092; p=0.765<br>F(1,20)=1.200; p=0.286<br>F(1,20)=8.249; p=0.009<br>F(1,20)=0.300; p=0.590<br>F(1,20)=1.759; p=0.200
**(b) Mechanical sensitivity before and after chronic nitroglycerin in orchidectomized wild-type or TRPM8 knockout mice supplemented with testosterone or vehicle**	3-way RM ANOVA	Day<br>Day*Genotype<br>Day*Nitroglycerin<br>Day*Genotype*Nitroglycerin<br>Genotype<br>Nitroglycerin<br>Genotype*Nitroglycerin	F(5,100)=104.609; p=0.000<br>F(5,100)=2.095; p=0.072<br>F(5,100)=5.188; p=0.000<br>F(5,100)=2.328; p=0.048<br>F(1,20)=4.721; p=0.042<br>F(1,20)=13.125; p=0.002<br>F(1,20)=0.978; p=0.335
**(c) Mechanical sensitivity in nitroglycerin-orchidectomized wild-type or TRPM8 knockout mice supplemented with testosterone or its vehicle, before and after AMTB**	3-way RM ANOVA	Day<br>Day*Genotype<br>Day*Nitroglycerin<br>Day*Genotype*Nitroglycerin<br>Genotype<br>Nitroglycerin<br>Genotype*Nitroglycerin	F(1,20)=0.004; p=0.949<br>F(1,20)=3.650; p=0.071<br>F(1,20)=5.379; p=0.031<br>F(1,20)=11.725; p=0.003<br>F(1,20)=8.014; p=0.010<br>F(1,20)=29.288; p=0.000<br>F(1,20)=3.768; p=0.066
**(d) Mechanical sensitivity in nitroglycerin-treated wild-type and TRPM8 knockout females before and after 1 mg/kg Testosterone or its vehicle**	2-way ANOVA	Time<br>Treatment Group<br>Time*Treatment Group	F(1,42)=0.5206; p=0.4746<br>F(2,42)=7.903; p=0.0012<br>F(2,42)=5.827; p=0.0058
**(e) Percentages of trigeminal neurons from wild-type and TRPM8KO animals responding Testosterone and WS12**	Mann-Whitney U	Genotype	p=0.0286
**(f) Size of calcium responses to testosterone in HEK293 cells naïve or transfected with murine or humanTRPM8**	Kruskal-Wallis followed by Mann Whitney U	Naïve vs. murine or human TRPM8	p<0.001

RM, Repeated Measures; ANOVA, Analysis of Variance; TRPM8, Transient Receptor Potential Melastatin 8; TRPM8KO, TRPM8 knockout; WS12, TRPM8 agonist (1R,2S,5R)-2-Isopropyl-N-(4-methoxyphenyl)-5-methylcyclohexanecarboxamide; AMTB, TRPM8 blocker, N-(3-aminopropyl)-2-{[(3-methylphenyl) methyl]oxy}-N-(2-thienylmethyl)benzamide hydrochloride; F, F-test; HEK293, Human Embrionic Kidney 293 cells.

Supplementary table 5. Statistical results for Figure 5.

	Statistical analysis	Result	
**(b) Outward current increase in hTRPM8 expressing HEK293 cells after 10pM testosterone application**	Shapiro-Wilk normality test	Basal<br>Testosterone 10pM<br>Testosterone+AMTB	W=0.74;p=0.024<br>W=0.91;p=0.375<br>W=0.866;p=0.252

	Kruskal-Wallis followed by Mann Whitney U	Basal vs Testos 10pM<br>Testos vs Testos+AMTB	p=0.017<br>p=0.009
**(d) Outward current increase in hTRPM8 expressing HEK293 cells after 10pM testosterone application independently from androgen receptor expression**	Shapiro-Wilk normality test	HEK293 WT<br>hTRPM8 + AR siARN<br>hTRPM8 + Scrambled siARN	W=0.74;p=0.015<br>W=0.94;p=0.642<br>W=0.89;p=0.334
	Kruskal-Wallis test followed by Mann Whitney U	HEK293 WT vs	HEK hTRPM8 + Scrambled siARN p=0.005<br>HEK hTRPM8 + AR siARN p=0.046
**(f) Testosterone dose-response curve in presence or not of AMTB**	Sigmoidal 4PL	Testosterone	LogIC50=-5.158<br>HillSlope=1.782<br>Degrees of Freedom=28<br>R squared=0.92
		Testosterone +AMTB	LogIC50=-4.473<br>HillSlope=1.718<br>Degrees of Freedom=7<br>R squared=0.82

AMTB, TRPM8 blocker, N-(3-aminopropyl)-2-{[(3-methylphenyl) methyl]oxy}-N-(2-thienylmethyl)benzamide hydrochloride, HEK293, Human Embrionic Kidney 293 cells.

Supplementary table 6. Statistical results for Supplementary Figure 1.

	Statistical analysis		Result
**Mechanical sensitivity 2h after nitroglycerin or vehicle treatments in males**	2-way RM ANOVA	Day<br>Nitroglycerin<br>Nitroglycerin*day	F(4,44)=2.583; p=0.050<br>F(1,11)=234.894; p=0.000<br>F(4,44)=2.028; p=0.132
**Mechanical sensitivity before and after chronic nitroglycerin or vehicle in males**	2-way RM ANOVA	Day<br>Nitroglycerin<br>Nitroglycerin*day	F(10,110)=19.076; p=0.000<br>F(1,11)=112.309; p=0.000<br>F(10,110)=12.921; p=0.000
**Mechanical sensitivity 2h after nitroglycerin or vehicle treatments in females**	2-way RM ANOVA	Day<br>Nitroglycerin<br>Nitroglycerin*day	F(4,40)=1.001; p=0.418<br>F(1,10)=209.975; p=0.000<br>F(4,40)=3.715; p=0.012
**Mechanical sensitivity before and after chronic nitroglycerin or vehicle in females**	2-way RM ANOVA	Day<br>Nitroglycerin<br>Nitroglycerin*day	F(10,100)=15.379; p=0.000<br>F(1,10)=213.597; p=0.000<br>F(10,100)=3.214; p=0.000

RM, Repeated Measures; ANOVA, Analysis of Variance; F, F-test.

Supplementary table 7. Statistical results for Supplementary Figure 2.

	Statistical analysis		Result	
**(c) Percentage of cultured trigeminal neurons responding to AITC**	2-way ANOVA	Sex<br>Treatment<br>Sex*Treatment	F(1, 16) =1.904;<br>F(1, 16)=46.77;<br>F(1, 16)=0.341;	p=0.187<br>p<0.001<br>p=0.567
**(d) Correlation between percentage of cells sensitive to AITC and size of response**	Linear regression	Percentage of AITC-sensitive cells vs size of response to AITC	r^2=0.5545;	p<0.001

ANOVA, Analysis of Variance; AITC, Allyl Isothiocyanate; F, F-test.

Supplementary table 8. Statistical results for Supplementary Figure 3.

	Statistical analysis	Result	
**(a) Percentage of cultured trigeminal neurons responding to WS12**	2-way ANOVA	Sex<br>Treatment<br>Sex*Treatment	F(1,16)= 0.037; p=0.850<br>F(1,16)=0.003; p=0.957<br>F(1,16)= 0.033; p=0.857

WS12, TRPM8 agonist (1R,2S,5R)-2-Isopropyl-N-(4-methoxyphenyl)-5-methylcyclohexanecarboxamide; ANOVA, Analysis of Variance; RM, Repeated Measures; F, F-test.

Supplementary table 9. Statistical results for Supplementary Figure 4.

	Statistical analysis	Result	
**(a) Mechanical sensitivity in nitroglycerin-treated wild-type and TRPM8 knockout females before and after 5 mg/kg WS12 or its vehicle**	2-way ANOVA	Time<br>Treatment Group<br>Time*Treatment Group	F(1,42)=0.8535; p=0.3609<br>F(2,42)=0.7703; p=0.4693<br>F(2,42)=1.482; p=0.2390
**(b) Mechanical sensitivity in nitroglycerin-treated wild-type and TRPM8 knockout females before and after 20 mg/kg WS12 or its vehicle**	2-way ANOVA	Time<br>Treatment Group<br>Time*Treatment Group	F(1,42)=1.687; p=0.2011<br>F(2,42)=4.337; p=0.0194<br>F(2,42)=1.447; p=0.2468
**(c) Mechanical sensitivity in nitroglycerin or vehicle-treated wild-type females before and after WS12 or its vehicle containing ethanol**	3-way RM ANOVA	Time<br>Time*WS12<br>Time*Nitroglycerin<br>Time*WS12*Nitroglycerin<br>WS12<br>Nitroglycerin<br>WS12*Nitroglycerin	F(1,19)=45.893; p=0.000<br>F(1,19)=2.226; p=0.152<br>F(1,19)=14.294; p=0.001<br>F(1,19)=3.929; p=0.062<br>F(1,19)=1.823; p=0.193<br>F(1,19)=78.423; p=0.000<br>F(1,19)=0.719; p=0.407

WS12, TRPM8 agonist (1R,2S,5R)-2-Isopropyl-N-(4-methoxyphenyl)-5-methylcyclohexanecarboxamide; ANOVA, Analysis of Variance; RM, Repeated Measures; F, F-test.

Supplementary table 10. Statistical results for Supplementary Figure 5.

	Statistical analysis	Result	
**(a) Testosterone and WS12 dose-response curves**	Sigmoidal 4PL	Testosterone	LogIC50=-5.158<br>HillSlope=1.782<br>Degrees of Freedom=28<br>R squared=0.92
		WS12	LogIC50=-0.303<br>HillSlope=0.867<br>Degrees of Freedom=18<br>R squared=0.93
**(b) AMTB dose-response curve vs 500nM WS12**	Sigmoidal 4PL	AMTB	LogIC50=-1.831<br>HillSlope=-0.933<br>Degrees of Freedom=14<br>R squared=0.84

WS12, TRPM8 agonist (1R,2S,5R)-2-Isopropyl-N-(4-methoxyphenyl)-5-methylcyclohexanecarboxamide; AMTB, TRPM8 blocker, N-(3-aminopropyl)-2-{[(3-methylphenyl) methyl]oxy}-N-(2-thienylmethyl)benzamide hydrochloride
