# Supplementary material for: TRPM8 contributes to sex dimorphism by promoting recovery of normal sensitivity in a mouse model of chronic migraine
Source: Nat Commun. 2022 Oct 22;13:6304. doi: 10.1038/s41467-022-33835-3 (PMC9588003; doi:10.1038/s41467-022-33835-3)
Supplement: Supplementary file 3 — Reporting Summary [file 41467_2022_33835_MOESM3_ESM.pdf]

Corresponding author(s): David Cabañero
Asia Fernández-Carvajal
Antonio Ferrer-Montiel

Last updated by author(s): Sep 14, 2022

Reporting Summary

Nature Portfolio wishes to improve the reproducibility of the work that we publish. This form provides structure for consistency and transparency in reporting. For further information on Nature Portfolio policies, see our Editorial Policies and the Editorial Policy Checklist.

Statistics

For all statistical analyses, confirm that the following items are present in the figure legend, table legend, main text, or Methods section.

n/a	Confirmed	
☐	☒	The exact sample size (*n*) for each experimental group/condition, given as a discrete number and unit of measurement
☐	☒	A statement on whether measurements were taken from distinct samples or whether the same sample was measured repeatedly
☐	☒	The statistical test(s) used AND whether they are one- or two-sided<br>*Only common tests should be described solely by name; describe more complex techniques in the Methods section.*
☐	☒	A description of all covariates tested
☐	☒	A description of any assumptions or corrections, such as tests of normality and adjustment for multiple comparisons
☐	☒	A full description of the statistical parameters including central tendency (e.g. means) or other basic estimates (e.g. regression coefficient) AND variation (e.g. standard deviation) or associated estimates of uncertainty (e.g. confidence intervals)
☐	☒	For null hypothesis testing, the test statistic (e.g. *F*, *t*, *r*) with confidence intervals, effect sizes, degrees of freedom and *P* value noted<br>*Give P values as exact values whenever suitable.*
☒	☐	For Bayesian analysis, information on the choice of priors and Markov chain Monte Carlo settings
☒	☐	For hierarchical and complex designs, identification of the appropriate level for tests and full reporting of outcomes
☒	☐	Estimates of effect sizes (e.g. Cohen's *d*, Pearson's *r*), indicating how they were calculated

Our web collection on statistics for biologists contains articles on many of the points above.

Software and code

Policy information about availability of computer code

Data collection	HCimage DIA software 1.0 (Hamamatsu Photonics K.K., Hamamatsu City, Japan), YASARA version 21.12.19 (YASARA Biosciences GmbH, Bio Prodict and WHAT IF Foundation), Patchmaster software v2x53 (HEKA Electronik, Dr. Schulze GmbH, Germany), ClustalO (1.2.4) at EBI (https://www.ebi.ac.uk/services).
Data analysis	IBM SPSS Statistics Version 25 (IBM Corporation, Armonk, NY, USA), GraphPad Prism 7.04 (GraphPad Software Inc., San Diego, CA, USA), Microsoft Excel 2019 MSO (Redmond, WA, USA), The PyMol Molecular Graphics System, version 2.5.0 (Schrödinger Inc, New York, NY, USA)

For manuscripts utilizing custom algorithms or software that are central to the research but not yet described in published literature, software must be made available to editors and reviewers. We strongly encourage code deposition in a community repository (e.g. GitHub). See the Nature Portfolio guidelines for submitting code & software for further information.

Data

Policy information about availability of data

All manuscripts must include a data availability statement. This statement should provide the following information, where applicable:

- Accession codes, unique identifiers, or web links for publicly available datasets
- A description of any restrictions on data availability
- For clinical datasets or third party data, please ensure that the statement adheres to our policy

All source data from the figures are available within the manuscript as a Source data file and results of statistical analyses are provided within the Supplementary Information file. Example dataset of calcium imaging (Figure 2CD) and analysis instructions have been deposited in Github: https://github.com/greferball/Ca_Imaging_NatComm_2022.git . All the calcium imaging datasets generated and analyzed during the study are available from the corresponding authors on request. Data used for computational analyses are available as supplementary information in the zip folder Computational_files.zip which contains instructions in a

readme.txt and scripts.

Field-specific reporting

Please select the one below that is the best fit for your research. If you are not sure, read the appropriate sections before making your selection.

☒ Life sciences ☐ Behavioural & social sciences ☐ Ecological, evolutionary & environmental sciences

For a reference copy of the document with all sections, see nature.com/documents/nr-reporting-summary-flat.pdf

Life sciences study design

All studies must disclose on these points even when the disclosure is negative.

Sample size	Sample size for behavioral and cellular studies was based on previous experience of the laboratory in similar experiments. For animal studies i.e. Cabañero et al., 2020 10.7554/eLife.55582, Célérier et al., 2006 10.1097/00000542-200603000-00023. For cellular studies i.e. Devesa et al., 2014 10.1073/pnas.1420252111, Journigan et al., 2021 10.1021/acsmedchemlett.1c00001.
Data exclusions	Results from calcium imaging were eliminated when basal line instability (standard deviation of basal values multiplied by 10) were superior to the fluorescence variation (peak value after positive control perfusion minus previous basal fluorescence values) elicited by positive control (KCl in neurons, Ionomicin in HEK293 and IMR90 cells). No other data were excluded.
Replication	Cellular experiments were reproduced at least three times. Consistent calcium imaging recordings could be achieved by using electronically-activated valves and a heat exchanger peltier for solution application. Each complete behavioural experiment with repeated mechanosensitivity measures was conducted once in different sets of animals, and the formalin tests were conducted in two different evaluation sets in males and in females. Experimental conditions of the repeated measures experiments were replicated across experiments containing groups of animals of 5-9 animals per condition: 4 experiments assessed mechanosensitivity after chronic nitroglycerin in wild type intact male mice and 8 experiments evaluated the effects of chronic nitroglycerin in wild type females. TRPA1 knockouts (male and female) were chronically treated with nitroglycerin in one experiment. Two different experiments assessed the effects of nitroglycerin in TRPM8 knockouts (2 each in males and females). Orchidectomized males were exposed to chronic nitroglycerin in 2 separate experiments. Testosterone replacement was assessed in one experiment containing two sets of testosterone-exposed mice. AMTB effects on mechanosensitivity were evaluated in two separate experiments and in the formalin test in males. The effects of different doses of WS12 dissolved in distinct vehicles were evaluated on females in three different mechanosensitivity experiments containing 2 sets of animals each. The effects of testosterone on female mechanosensitivity were evaluated in one experiment also with 2 sets of animals. Electrophysiological experiments assessing the function of TRPM8 in HEK293 were repeated twice, and the Knockdown of the androgen receptor in HEK293 cells heterologously expressing TRPM8 was assayed twice and corroborated each time through western blot.
Randomization	For the behavioural studies on mechanosensitivity, animals of same sex and/or genotype were evaluated for baseline sensitivity and homogeneous groups were established according to this parameter. Afterwards, treatments were randomly allocated in the different groups whenever applicable. For the formalin tests, the animals were randomly allocated in the different groups. Mice used to obtain samples for the cellular studies were of similar age between treatment groups but were randomly assigned to the treatments (8 to 12 week-old mice).In the cellular experiments the treatments applied were allocated randomly to the cells plates available at the time of the experiments.
Blinding	For the behavioral experiments, researchers were blinded for the studied experimental condition (nitroglycerin treatment, genotype) during the behavioral assay whenever the experimental design allowed this possiblity (e.g. not blinded for a given drug treatment in within-subject design experiments assessing the effect of such drug). For the cellular studies samples were labeled with the experimental condition in each experiment . For the Androgen Receptor knockdown of the HEK293 cells the experimenters were blinded for the siRNA used. Blinding in the other cellular experiments was not possible due to the availability of individual researchers for the preparation and application of compounds.

Reporting for specific materials, systems and methods

We require information from authors about some types of materials, experimental systems and methods used in many studies. Here, indicate whether each material, system or method listed is relevant to your study. If you are not sure if a list item applies to your research, read the appropriate section before selecting a response.

Materials & experimental systems

n/a	Involved in the study	
☐	☒	Antibodies
☐	☒	Eukaryotic cell lines
☒	☐	Palaeontology and archaeology
☐	☒	Animals and other organisms
☒	☐	Human research participants
☒	☐	Clinical data
☒	☐	Dual use research of concern

Methods

n/a	Involved in the study	
☒	☐	ChIP-seq
☒	☐	Flow cytometry
☒	☐	MRI-based neuroimaging

Antibodies

Antibodies used	Primary<br>Rabbit anti-MAP (17490-1-AP, LabClinics, Barcelona, Spain). Lot. GR3336715-4<br>Mouse anti-CGRP (AB81887, Abcam, Cambridge, UK). Lot. 00079896<br>Mouse anti-Androgen Receptor (sc-7305, Santa Cruz Biotechnology, Dallas, TX, USA)<br>Rabbit anti-β-tubulin (10094-1-AP, Proteintech, Manchester, UK)<br><br>Secondary<br>Goat anti-rabbit Alexa 488 (A11034, Thermo Fisher Scientific) Lot. 1298480<br>Goat anti-mouse Alexa 568 (A11031, Thermo Fisher Scientific) Lot. 2300933<br>anti-mouse IgG-HRP (A4416, Sigma)<br>anti-rabbit IgG-HRP (A0545, Sigma)
Validation	The anti-CGRP antibody has been validated in inmunohistochemical studies using CGRP knockout mice (Chen et al., Neuron 2018 doi: 10.1016/j.neuron.2018.09.032).Anti-CGRP was validated by manufacturer for immunofluorescence (DRG neurons). This antibody was used in 97 publications, e.g. PMID 31446225, 29588412.<br>Anti-MAP was validated by manufacturer for western blot (mouse brain tissue, rat brain tissue and SH-SY5Y cells), immunohistochemistry (mouse brain tissue, rat brain tissue, human gliomas tissue, mouse cerebellum tissue and human brain) and immunofluorescence (iPS cells, rat brain tissue and mouse brain tissue). This antibody was used to identify neurons in 144 papers, e.g. PMID 30624794.<br>Anti-AR was validated by manufacturer for western blot (ZR-75-1, MCF7 and LNCap cells) and immunofluorescence (T-47D cells). It was used in 566 publications, e.g. PMID 35402240. We have validated the Androgen receptor antibody through siRNA knockdown of the Androgen receptor using androgen receptor siRNA (ThermoFisher, Ambion, s1538).<br>Anti-β-tubulin was validated by manufacturer for western blot (HEK293 cells, U-251 cells and mouse kidney tissue), immunohistochemistry (rat brain tissue, rat testis tissue, mouse brain tissue, human colon tissue and human cerebellum tissue), immunoprecipitation (mouse brain tissue) and immunofluorescence (HepG2 cells). It was used in 461 publications, e.g. PMID 31150838.

Eukaryotic cell lines

Policy information about cell lines

Cell line source(s)	Human embryonic kidney 293 cells (HEK293) were originally obtained from Prof. Belmonte Laboratory (Instituto de Neurociencias, San Juan, Alicante, Spain). HEK293 cells were obtained from the European Collection of Authenticated Cell Cultures (85120602).<br>Human embryonic kidney 293 cells (HEK293) TRPM8 permanently transfected cells were obtained from Prof. Belmonte Laboratory (Instituto de Neurociencias, San Juan, Alicante, Spain).<br>IMR90 fibroblast-like cells (CCL-186 ATCC, Virginia, USA).
Authentication	HEK293 cells are routinely used in the laboratory for biochemical, electrophysiological and calcium imaging studies and have been used for more than 10 years. They were checked for usual morphology for the current studies. HEK293 cells transfected with TRPA1 were tested with TRPA1 agonist AITC and cells transfected with TRPM8 were checked with TRPM8 agonist WS12.<br>Authentication of commercial IMR90 cells was done based on fibroblast morphology and responses to TRPA1 stimulation, typical of these cells as demonstrated in Tonello et al in Br.J.Pharmacol in 2017 (doi: 10.1111/bph.13652).
Mycoplasma contamination	Regular mycoplasma contamination tested negative.
Commonly misidentified lines<br>(See ICLAC register)	None.

Animals and other organisms

Policy information about studies involving animals; ARRIVE guidelines recommended for reporting animal research

Laboratory animals	8-15 week-old male and female C57BL/6J mice (Envigo, Horst, The Netherlands), and male and female TRPM8 knockouts (Bautista et al., 2007 doi: 10.1038/nature05910) and TRPA1 knockouts (Kwan et al., 2006 doi: 10.1016/j.neuron.2006.03.042) all in C57BL/6J background.
Wild animals	No wild animals were used in the study.
Field-collected samples	No field collected samples were used in the study.
Ethics oversight	All experimental procedures were approved by the Animal Care and Use Committees of Universidad Miguel Hernández and the regional government (Conselleria de Agricultura, Desarrollo Rural, Emergencia Climática y Transición Ecológica from Generalitat Valenciana, Spain; Code: 2018/VSC/PEA/0250-3) and were conducted according to the ethical principles of the International Association for the Study of Pain (IASP) for the evaluation of pain in conscious animals, the European Parliament and the Council Directive (2010/63/EU) and the Spanish law (RD 53/2013).

Note that full information on the approval of the study protocol must also be provided in the manuscript.
